# Cells bearing Fc receptors in human malignant solid tumours.

**DOI:** 10.1038/bjc.1982.34

**Published:** 1982-02

**Authors:** J. L. Svennevig, T. R. Andersson

## Abstract

**Images:**


*Br. J. Cancer* (1982) **45**, 201

### CELLS BEARING Fc RECEPTORS IN HUMAN MALIGNANT SOLID TUMOURS

J.-L. SVENNEVIG AND T. R. ANDERSSON

*From the Institute for Experimental Medical Research, University of Oslo, Ullevål Hospital, Oslo, Norway*

Received 27 July 1981 Accepted 6 October 1981

**Summary.—Fc-receptor-bearing cells forming EA rosettes with antibody-coated human erythrocytes (Ripley) were studied in cell suspensions and in purified preparations of mononuclear cells (MC) from 20 human malignant tumours.**

**The EA rosettes were studied in preparations made by cytocentrifugation and the rosette-forming cells identified by their nonspecific-esterase activity and phagocytic capacity.**

**Fc receptors were found on 16 ± 20% of all cells in the primary cell suspensions. Significantly more tumour-infiltrating lymphocytes had detectable Fc receptors (33 ± 18%) than did peripheral-blood lymphocytes in cancer patients (19 ± 8%) and normal control subjects (14 ± 6%). There was a significant correlation between the proportion of lymphocytes lacking T and B markers (null cells) and the proportion of lymphocytes with Fc receptors.**

**Fc receptors were also found on most tumour-infiltrating macrophages, on some T lymphocytes and polymorphonuclear cells and on a smaller percentage of the tumour cells.**

**The significance of the Fc receptor and its usefulness as a marker of "host infiltration" into the tumours is discussed.**

HUMAN SOLID NEOPLASMS contain various amounts of white blood cells which are believed to represent an immune response against the malignant cells. Most authors find the degree of mononuclear cell (MC) infiltration in cancer positively correlated with improved prognosis (Underwood, 1974).

We have previously examined MC isolated from different human tumours and demonstrated lymphocytes with T and B markers in practically all tumours (Svennevig *et al.*, 1978, 1979). However, a large proportion of the lymphocytes lacked T and B markers, and were thus considered as "null cells". A large proportion of macrophages was also demonstrated in the tumours (Svennevig & Svaar, 1979).

In peripheral blood, a third population of lymphocytes lacking markers for both T and B lymphocytes has been shown to carry receptors for the Fc portion of IgG (Frøland & Natvig, 1973). The present study was carried out in order to examine the presence of the Fc receptor on MC harvested from 20 human neoplasms, and whether the Fc marker may be used to measure "host infiltration" (Kerbel & Pross, 1976) into the tumours.

### MATERIALS AND METHODS

*Patients and specimens.*—Fresh biopsy specimens weighing 0·5–6·0 g, were taken as a representative sample from 20 tumours removed by operation. No blood transfusions or irradiation had been given previously. All visible fat and connective tissue was cut away and the biopsy specimens were washed

Correspondence: Dr Jan-L. Svennevig, Department of Surgery, Rikshospitalet, Oslo, Norway.

thoroughly to remove contaminating white blood cells.

*Tumour-cell suspensions.*—The tumour tissue was disaggregated mechanically by cutting small pieces into phosphate-buffered saline (PBS) and squeezing them through fine metal meshes followed by magnetic stirring of the suspension for 30 min. Non-disaggregated tissue fragments were removed by filtering the cell suspension through double-layered gauze.

The degree of contaminating peripheral-blood cells was calculated from the lymphocyte/erythrocyte ratio in both tumour-cell suspension and peripheral blood (Svennevig *et al.*, 1978) and viability was assessed by trypan-blue exclusion.

*Isolation and characterization of MC.*—The tumour-cell suspensions were layered on Lymphoprep (Nyegaard & Co. Oslo, Norway) and centrifugated at 400 *g* for 40 min at room temperature. The MC were harvested from the interface, washed twice with PBS, and examined for ability to form E rosettes with untreated sheep red blood cells (SRBC) as a marker for T lymphocytes, for the presence of surface-bound Ig as a marker of B lymphocytes, for ability to ingest latex particles and to form EA rosettes with human $OR_1R_2$ erythrocytes coated with anti-CD Ripley.

*Removal of adherent cells.*—In 10 cases the MC were resuspended in medium RPMI 1640 supplemented with 20% foetal calf serum and incubated overnight in Falcon culture dishes to remove adherent cells. Both the monocyte/macrophage-depleted lymphocyte fraction and the adherent cells were then examined for cell markers as described above.

*Cytocentrifuge preparations.*—Fixed preparations were made from all suspensions by cytocentrifugation (Cytospin, Shandon-Elliott) at 600 rev/min. The E- and EA-rosetting cells were stained with May–Grünwald–Giemsa and with α-naphthyl acetate, and examined for non-specific-esterase activity and phagocytic capacity. Technical details concerning the use of cell markers and esterase techniques have been given in an earlier report (Andersson & Svennevig, 1981). Monocytes and macrophages are stained diffusely red by these techniques whereas distinct dots or a scattered cytoplasm reaction is seen in T lymphocytes.

*Tumour cells.*—Malignant cells from all 20 tumours were recovered from the bottom of the centrifuge tubes following the isolation of MC, and then washed and examined for EA-rosetting cells, which were further characterized in cytocentrifuge preparations.

*Peripheral-blood cells.*—MC were isolated from peripheral blood from all 20 cancer patients and from 20 healthy controls and examined for T and B markers, phagocytosis and ability to form EA rosettes with anti-CD Ripley-coated human erythrocytes. In 10 patients and 10 control subjects, lymphocytes and monocytes were further separated by adherence to plastic.

*Statistics.*—All data are given as mean ± s.d. and the *t*-test was used for calculation of probabilities.

### RESULTS

### *Isolation of MC from tumour tissue*

Twenty tumours were examined. Two cell suspensions were excluded because of contamination by PBL; it was calculated that 5–10% of the lymphocytes could have come from peripheral blood. The suspensions from the remaining 18 tumours contained 3(±)3% lymphocytes, 1 ± 1% plasma cells, 6 ± 6% macrophages and 6 ± 5% polymorphonuclear cells (PMN) (Table I). From the erythrocyte/lymphocyte ratio it was calculated that less than 1% of the lymphocytes in these suspensions came from peripheral blood.

A sufficient number of MC ($0{\cdot}3–7{\cdot}8 \times 10^6$/g tumour tissue) to allow the use of cell markers could be isolated from 15 out of 18 tumour-cell suspensions. The purified preparations contained 66 ± 22% MC. Recovery was 57 ± 29% for lymphocytes and 24 ± 34% for macrophages, when the initial number of cells in the primary cell suspensions was considered. More than 90% of the MC excluded trypan blue, while 60 ± 29% of the tumour cells were viable. After removal of adherent cells, the lymphocyte-enriched fraction consisted of 70 ± 18% lymphocytes, while 79 ± 16% of all adherent cells had the properties of macrophages; *i.e.* they were phagocytic and exhibited a diffuse esterase activity.

### *Cell markers*

Fc-receptor-bearing cells were found in the primary cell suspensions from all

TABLE I.—*Relative content of tumour infiltrating lymphocytes (TIL), macrophages (TIM) and polymorphonuclear cells (PMN) in cell suspensions from 20 human neoplasms (mean and range)*

| No. of cases | % TIL | % TIM | % PMN |
|---|---|---|---|
| 5 Carcinoma of the colon | 3 (2–6) | 3 (1–5) | 4 (3–5) |
| 4 Carcinoma of the stomach | 2 (0·5–5) | 4 (3–7) | 5 (0–10) |
| 6 Carcinoma of the lung* | 4 (1–12) | 13 (3–19) | 8 (0–22) |
| 1 Sarcoma of the thoracic wall | 0 | 2 | 6 |
| 1 Carcinoma of the breast* | — | — | — |
| 3 Carcinoma of the rectum | 3 (1–5) | 2 (0·5–4) | 6 (2–12) |
| Mean ± s.d. | 3 ± 3 | 6 ± 6 | 6 ± 5 |

* Data not given in 2 cases due to contamination by PBL.

TABLE II.—*Subpopulations of lymphocytes in tumour tissue and peripheral blood (mean and range)*

| No. of cases | % E | % sIg | % null | % EA |
|---|---|---|---|---|
| 15 TIL | 49 (6–80) | 13 (4–30) | 38 (12–85) | 33 (10–69) |
| 20 PBL, cancer patients | 67 (40–85) | 11 (6–18) | 22 (5–50) | 19 (7–35) |
| 20 PBL, normal controls | 73 (54–86) | 12 (6–18) | 15 (1–34) | 14 (5–24) |

tumours; 16 ± 20% of all nucleated cells formed EA rosettes.

Of the isolated tumour-infiltrating lymphocytes (TIL), 49 ± 20% formed spontaneous rosettes with SRBC, and 13 ± 6% had membrane-bound Ig, while 38 ± 22% lacked T and B markers (null cells). The relative proportion of null lymphocytes was significantly higher than for PBL in cancer patients and normal controls ($P = 0{\cdot}01$) (Table II).

A significantly higher percentage of TIL (33 ± 18%) had detectable Fc receptors (Fig. 1) than did the PBL in cancer patients (19 ± 8%) and normal controls (14 ± 6%), and there was a good correlation between the relative number of null cells in the tumours and the number of lymphocytes with Fc receptors ($P = 0{\cdot}01$) (Table III). Comparable data were obtained whether cytocentrifuge preparations from MC suspensions or monocytes-depleted lymphocyte suspensions were used. Of the EA-rosetting TIL, 15 ± 7% had esterase-positive dots characteristic of T lymphocytes, which did not differ significantly from the results for PBL in the patients (11 ± 5%) and normal controls (10 ± 6%).

There was no significant difference in

TABLE III.—*Cell markers on lymphocytes isolated from 15 tumour-cell suspensions*

| | % E | % sIg | % null | % EA |
|---|---|---|---|---|
| Bronchial carcinoma | 22 | 12 | 66 | 60 |
| | 43 | 14 | 43 | 42 |
| | 50 | 9 | 41 | 40 |
| | 50 | 16 | 34 | 12 |
| Carcinoma of the stomach | 54 | 14 | 32 | 38 |
| | 57 | 20 | 23 | 32 |
| | 60 | 13 | 27 | 25 |
| Carcinoma of the colon | 64 | 8 | 28 | 18 |
| | 80 | 8 | 12 | 10 |
| | 17 | 4 | 79 | 69 |
| Carcinoma of the rectum | 46 | 30 | 24 | 35 |
| | 75 | 12 | 13 | 14 |
| | 6 | 9 | 85 | 56 |
| | 56 | 16 | 28 | 33 |
| | 50 | 8 | 42 | 17 |
| Mean | 49 | 13 | 38 | 33 |

FIG. 1.—Two EA-rosetting lymphocytes from a bronchial carcinoma. Cytocentrifuge preparation. May–Grünwald–Giemsa staining. × 1250.

FIG. 2.—Esterase-positive, phagocytic cell (macrophage) forming an EA rosette. Cytocentrifuge preparation. α-Naphthyl acetate esterase and Giemsa staining. × 1250.

FIG. 3.—Neutrophilic granulocyte forming an EA rosette. Technique as Fig. 2.

FIG. 4.—Esterase-negative, non-phagocytic tumour cells from a colon carcinoma, binding anti-CD Ripley-coated human erythrocytes to their surface. Technique as Fig. 2.

Fc receptors on peripheral-blood monocytes from cancer patients ($82 \pm 12\%$) and normal controls ($74 \pm 14\%$), and a similar percentage of tumour-infiltrating macrophages formed EA rosettes ($82 \pm 13\%$) (Fig. 2).

Both neutrophilic and eosinophilic granulocytes were capable of forming EA rosettes, regardless of whether they were isolated from peripheral blood ($36 \pm 28\%$) or cancer tissue ($36 \pm 17\%$) (Fig. 3).

A small percentage ($9 \pm 15\%$) of esterase-negative, non-phagocytic cells morphologically similar to malignant tumour cells from 19/20 tumours formed typical EA rosettes (Fig. 4). However, EA rosettes formed by tumour cells were less stable and often difficult to fixate by cytocentrifugation.

### DISCUSSION

Fc receptors have previously been demonstrated in both human (Tønder & Thunold, 1973; Tønder *et al.*, 1974; Wood & Gollahon, 1977; Wesenberg, 1978) and experimental tumours (Kerbel & Pross, 1976; Haskill, 1977; Thomson *et al.*, 1979). However, there have been conflicting results on the identity of the receptor-bearing cells. Some previous reports have established that most Fc receptor-bearing cells within tumours were macrophages (Kerbel & Pross, 1976, Wood & Gollahon, 1977) and the Fc receptor has thus been caused as an index of host infiltration into the tumours (Kerbel & Pross, 1976). Other authors have claimed the presence of Fc receptors on tumour cells also (Tønder *et al.*, 1978; Biran *et al.*, 1979) and on a large proportion of TIL (Häyry & Tøtterman, 1978; Vose *et al.*, 1977). In malignant melanoma, the largest population of infiltrating cells were esterase-negative, non-phagocytic, non-T and non-B cells with Fc receptors (Roubin *et al.*, 1975).

In the present study, MC were isolated from tumour-cell suspensions by the widely used method of gradient separation, initially described by Bøyum (1968). Although many macrophages were lost, a high degree of purity and cell yield could be obtained for tumour lymphocytes, probably due to the use of fresh biopsy material, simple and fast procedures, a high degree of dilution of all cell suspensions and by reducing all washing procedures to a minimum. Unsuccessful separation due to cell aggregation (Svennevig *et al.*, 1978) could be prevented by removing non-disaggregated material and cell debris from the primary cell suspensions before the isolation procedure. By comparing cell suspensions with tissue sections from human tumours, we have previously demonstrated a correlation in respect of the inflammatory cells, indicating that tumour-cell suspensions, despite cell loss and cell damage, may reflect the real situation within the tumours (Svennevig & Holter, 1981).

Human $OR_1R_2$ erythrocytes coated with anti-CD Ripley have been shown to react with Fc receptors on null lymphocytes, some T lymphocytes, monocytes and PMN, while Fc receptors on B cells were not detected by this assay (Shaw *et al.*, 1979, Andersson & Svennevig, 1981). In the present study the superimposition of several markers in cytopreparations (Ranki *et al.*, 1976) demonstrated Fc receptors on at least 4 cell types: lymphocytes, macrophages, PMN and tumour cells. The study also demonstrated that the problem of enumerating rosettes in preparations containing more than 1 cell type can be overcome by identifying the rosetting cells in preparations fixed after cytocentrifugation. This is only possible when working with strong and stable rosettes. Cytocentrifuge fixation of rosettes using other types of indicator cells, such as antibody-coated chicken or sheep erythrocytes, has been less successful in our hands.

By correlating the percentages of each cell type carrying Fc receptors with the relative content of the cell populations in the primary cell suspensions, it may be calculated that only 50% of the EA rosettes were formed by white blood cells, while 50% were formed by tumour cells. Thus the Fc receptor should not be used as the sole marker for host infiltration into solid human tumours.

Lymphocytes lacking T and B markers represent only a small proportion in normal control subjects. However, in many conditions, such as progressing cancer, an increased proportion of null lymphocytes is found. This population may even dominate in the lymphocyte response at the tumour site (Häyry & Tøtterman, 1978; Svennevig *et al.*, 1978; Svennevig & Holter, 1981). The present study demonstrated a good correlation between the percentage of null lymphocytes and Fc-receptor-bearing lymphocytes in the tumours. The results confirm our previous findings of low percentages of T lymphocytes in some tumours, though the proportion in peripheral blood was within the normal range (Svennevig & Holter, 1981). The present study extends previous findings by demonstrating that most non-T lymphocytes carry receptors for the Fc portion of IgG.

The demonstration of an increased portion of Fc-receptor-bearing cells may reflect a real increase in the number of null cells, immature T cells (Balch *et al.*, 1980; Chiao *et al.*, 1980) or T suppressor cells (Ferranini *et al.*, 1980). Although Fc-receptor-bearing lymphocytes from peripheral blood may act as effector cells in antibody-dependent cytotoxicity (Dobloug *et al.*, 1980), no cytotoxic activity has been demonstrated for TIL (Vose *et al.*, 1977), and recent investigations have also failed to demonstrate any NK-cell effect (Moore & Vose, 1981). It is interesting, however, that an increased suppressor-cell activity has been demonstrated in human tumours (Vose & Moore, 1979). Non-specific Ig may also bind to Fc receptors of both target and effector cells (MacSween & Eastwood, 1980) and thereby hinder the immune reaction at the tumour site.

The present study was supported by grants from Anders Jahre's Fund for the Promotion of Science. The authors are grateful to Miss Lise Dyrkoren and Mr Roger Fane for their skilful technical assistance.

REFERENCES

Andersson, T. R. & Svennevig, J.-L. (1981) Which Fc receptor-bearing cells are detected with the Ripley assay? *Clin. Exp. Immunol.*, **44**, 167.

Balch, C. M., Ades, E. W., Loken, M. R. & Shore, S. L. (1980) Human "null" cells mediating antibody-dependent cellular cytotoxicity express T lymphocyte differentiation antigens. *J. Immunol.*, **124**, 1845.

Biran, H., Mavligit, G. M. & Moake, J. L. (1979) Receptor sites for complement and for immune complexes on human nonhemopoietic tumor cells. *Cancer*, **44**, 131.

Bøyum, A. (1968) Separation of leukocytes from blood and bone marrow. *Scand. J. Clin. Invest.* Suppl., **97**.

Chiao, J. W., Fried, J., Arlin, Z. A., Freidag, W. B. & Good, R. A. (1980) Delineation of the development of T lymphocytes from leukemic null lymphocytes upon induction by conditioned medium. *Cell Immunol.*, **51**, 331.

Dobloug, J. H., Thorsteinsson, L., Førre, Ø., Mellbye, O. J. & Natvig, J. B. (1980) Erythrocytes coated with anti-Rh Ripley react with both the T and the non-T lymphocytes active in antibody-dependent cell-mediated cytotoxicity. *Clin. Immunol. Immunopathol.*, **17**, 102.

Ferrarini, M., Cadoni, A., Franzi, A. T. & 4 others (1980) Ultrastructure and biochemistry of human peripheral blood lymphocytes. Similarities between the cells of the third population and $T_G$ lymphocytes. *Eur. J. Immunol.*, **10**, 562.

Frøland, S. S. & Natvig, J. B. (1973) Identification of three different human lymphocyte populations by surface markers. *Transplant Rev.*, **16**, 114.

Häyry, P. & Tøtterman, T. H. (1978) Cytological and functional analysis of inflammatory infiltrates in human malignant tumors. I. Composition of the inflammatory infiltrates. *Eur. J. Immunol.*, **8**, 866.

Haskill, J. S. (1977) ADCC effector cells in a murine adenocarcinoma. I. Evidence for blood-bone-marrow-derived monocytes. *Int. J. Cancer*, **20**, 432.

Kerbel, R. S. & Pross, H. F. (1976) Fc receptor-bearing cells as a reliable marker for quantitation of host lymphoreticular infiltration of progressively growing solid tumors. *Int. J. Cancer*, **18**, 432.

Moore, M. & Vose, B. M. (1981) Extravascular natural cytotoxicity in man: Anti-K 562 activity of lymph node and tumour-infiltrating lymphocytes. *Int. J. Cancer*, **27**, 265.

MacSween, J. M. & Eastwood, S. L. (1980) Immunoglobulins associated with human tumours *in vivo*: IgG concentrations in eluates of colonic carcinomas. *Br. J. Cancer*, **42**, 503.

Ranki, A., Tøtterman, T. H. & Häyry, P. (1976) Identification of resting human T and B lymphocytes by acid α-naphthyl acetate esterase staining combined with rosette formation with *Staphylococcus aureus* strain Cowan 1. *Scand. J. Immunol.*, **5**, 1129.

Roubin, R., Césarini, J.-P., Fridman, W. H., Pavie-Fischer, J. & Peter, H. H. (1975) Characterization of the mononuclear cell infiltrate in human malignant melanoma. *Int. J. Cancer*, **16**, 61.

SHAW, G. M., LEVY, P. C. & LOBUGLIO, A. F. (1979) Re-examination of the EA rosette assay (Ripley) for Fc receptor leucocytes. *Clin. Exp. Immunol.*, **36**, 496.

SVENNEVIG, J.-L., CLOSS, O., HARBOE, M. & SVAAR, H. (1978) Characterization of lymphocytes isolated from non-lymphoid human malignant tumours. *Scand. J. Immunol.*, **7**, 487.

SVENNEVIG, J.-L. & HOLTER, J. (1981) The local cell response to human lung carcinomas. *Acta Pathol. Microbiol. Scand. Sect. A*, **89**, 147.

SVENNEVIG, J.-L., LØVIG, M. & SVAAR, H. (1979) Isolation and characterization of lymphocytes and macrophages from solid, malignant human tumours. *Int. J. Cancer*, **23**, 626.

SVENNEVIG, J.-L. & SVAAR, H. (1979) Content and distribution of macrophages and lymphocytes in solid, malignant human tumours. *Int. J. Cancer*, **24**, 754.

THOMSON, A. W., CRUICKSHANK, N. & FOWLER, E. F. (1979) Fc receptor-bearing and phagocytic cells in syngeneic tumours of *C. parvum* and carrageenan-treated mice. *Br. J. Cancer*, **39**, 598.

TØNDER, O., MORSE, P. A., JR & HUMPHREY, L. J. (1974) Similarities of Fc receptors in human malignant tissue and normal lymphoid tissue. *J. Immunol.*, **113**, 1162.

TØNDER, O. & THUNOLD, S. (1973) Receptors for immunoglobulin Fc in human malignant tissues. *Scand. J. Immunol.*, **2**, 207.

TØNDER, O., KRISHNAN, E. C., MORSE, P. A., JR, JEWELL, W. R. & HUMPHREY, L. J. (1978) Localization of Fc receptors in human and rat malignant tissues. *Acta Pathol. Microbiol. Scand. Sect. C*, **86**, 173.

UNDERWOOD, J. C. E. (1974) Lymphoreticular infiltration in human tumours: Prognostic and biological implications: A review. *Br. J. Cancer*, **30**, 538.

VOSE, B. M. & MOORE, M. (1979) Suppressor cell activity of lymphocytes infiltrating human lung and breast tumours. *Int. J. Cancer*, **24**, 579.

VOSE, B. M., VANKY, F. & KLEIN, E. (1977) Human tumour-lymphocyte interactions *in vitro*. V. Comparison of the reactivity of tumour-infiltrating, blood and lymph-node lymphocytes with autologous tumour cells. *Int. J. Cancer*, **20**, 895.

WESENBERG, F. (1978) Fc$\gamma$ receptors and IgG associated with human malignant tumours. *Acta Pathol. Microbiol. Scand. Sect. C*, **86**, 259.

WOOD, G. W. & GOLLAHON, K. A. (1977) Detection and quantitation of macrophage infiltration into primary human tumours with the use of cell-surface markers. *J. Natl. Cancer Inst.*, **59**, 1081.


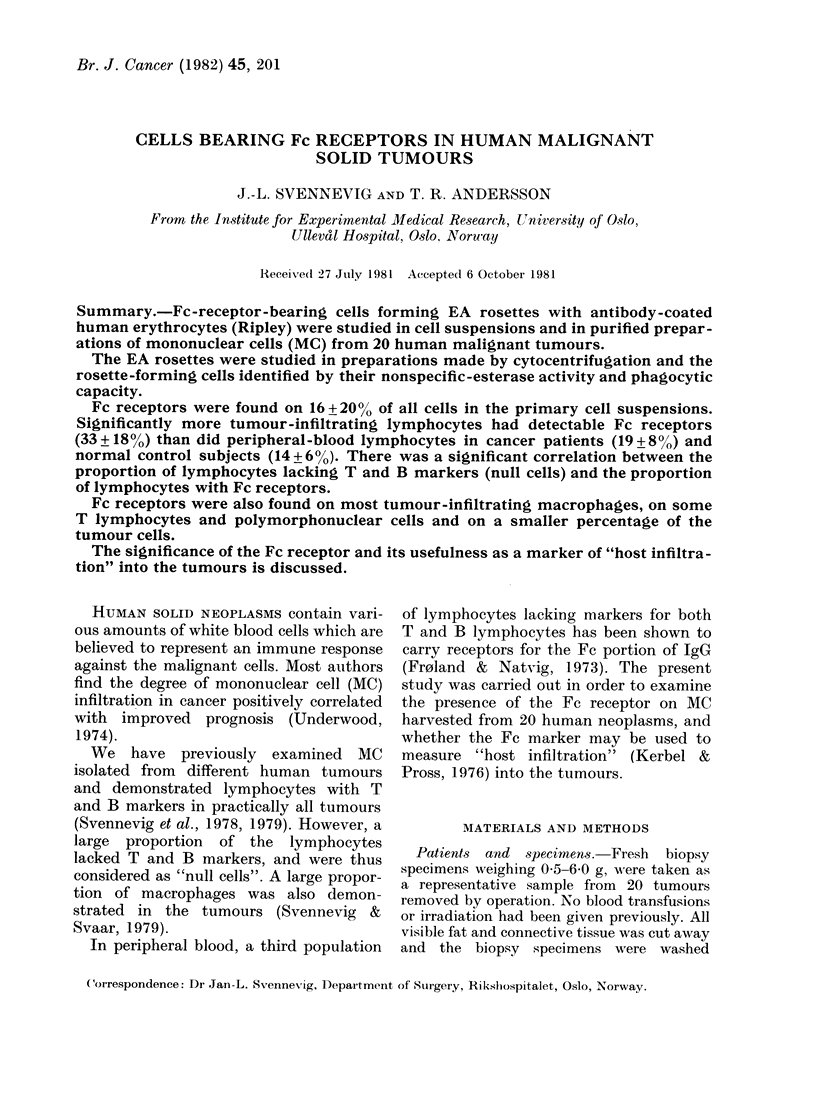

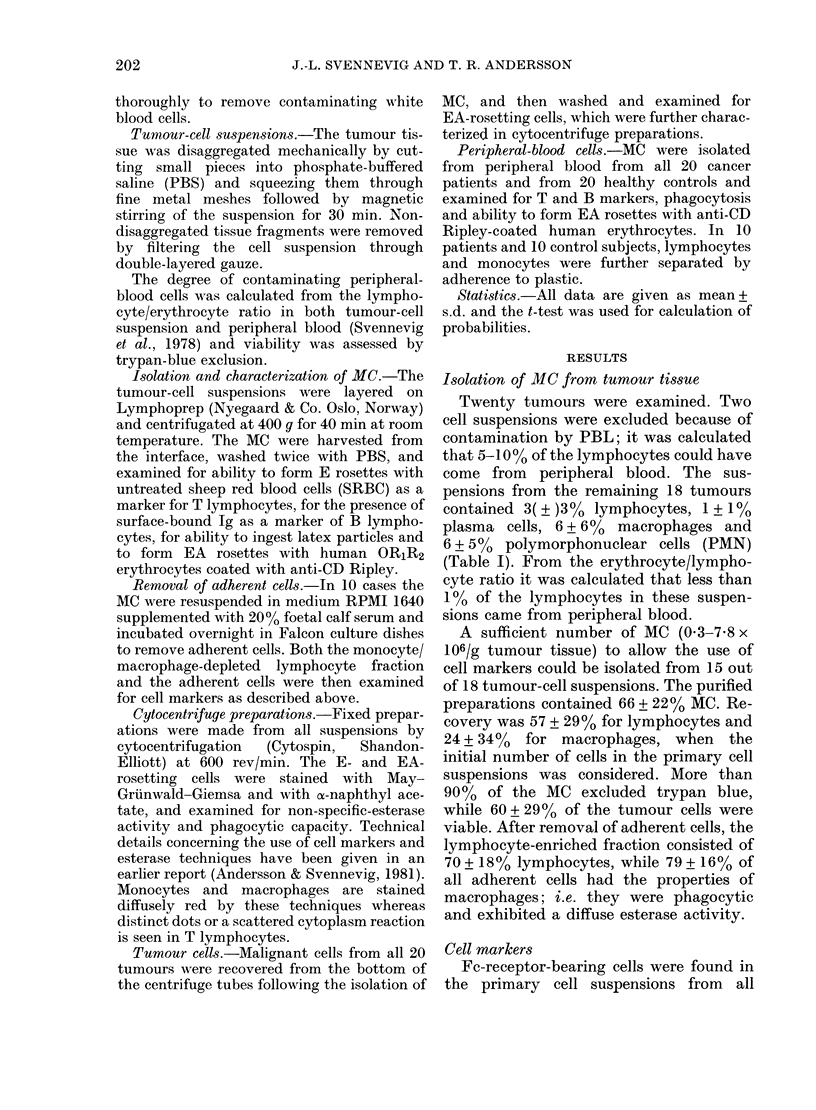

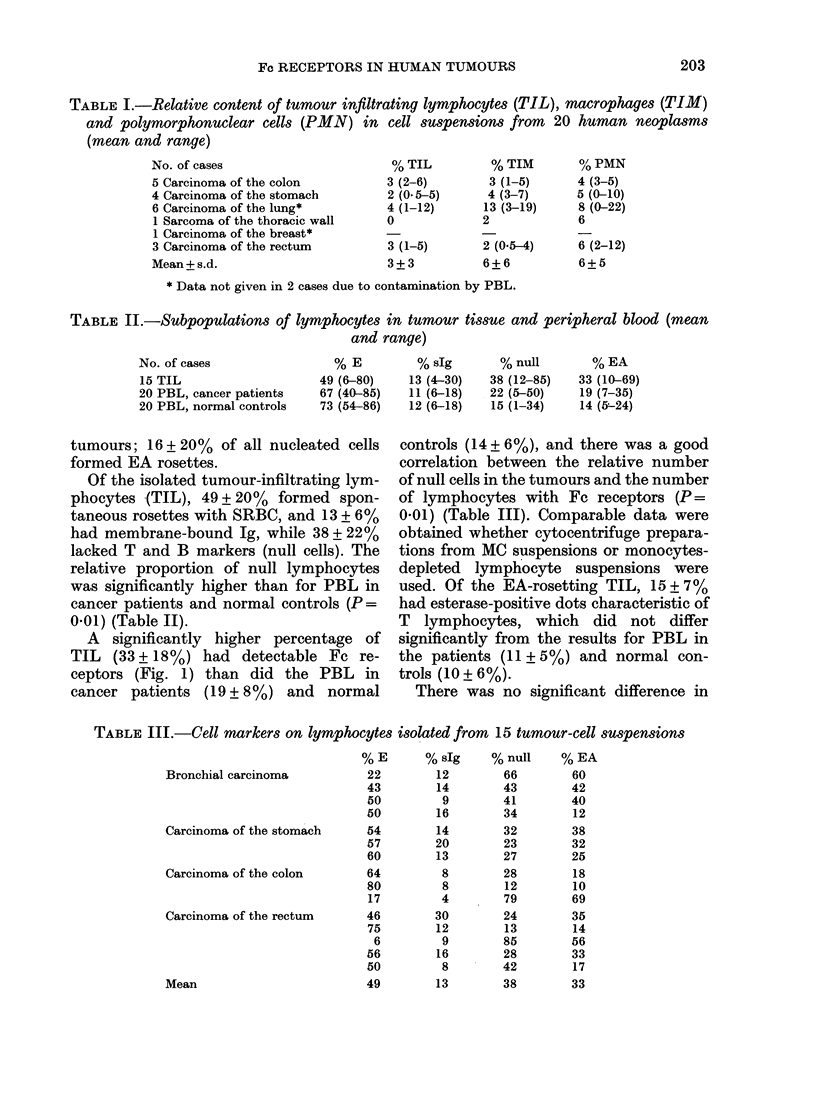

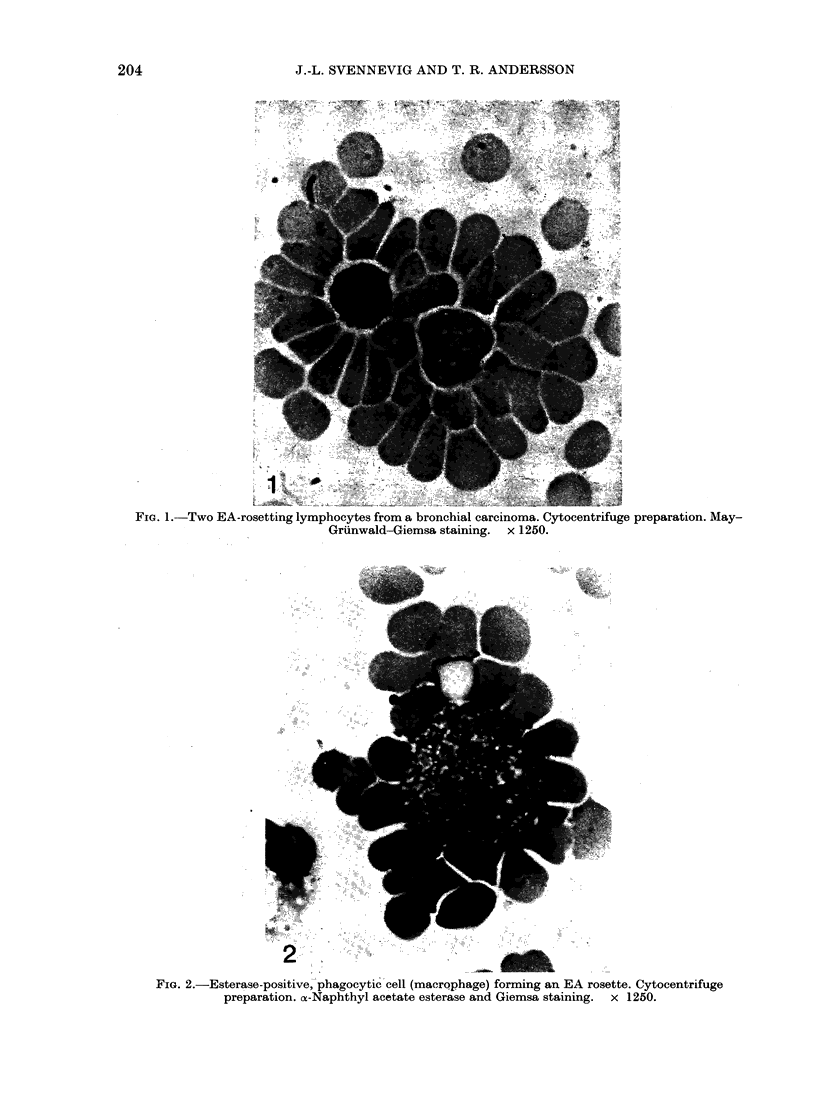

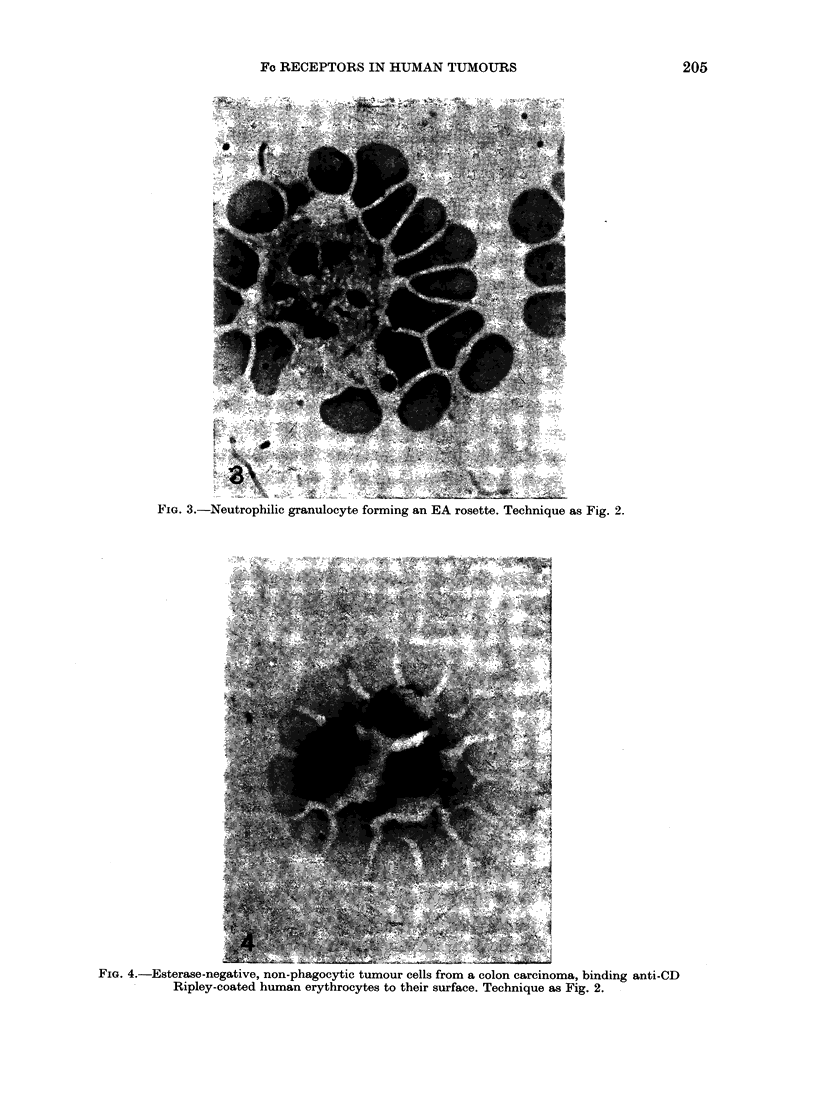

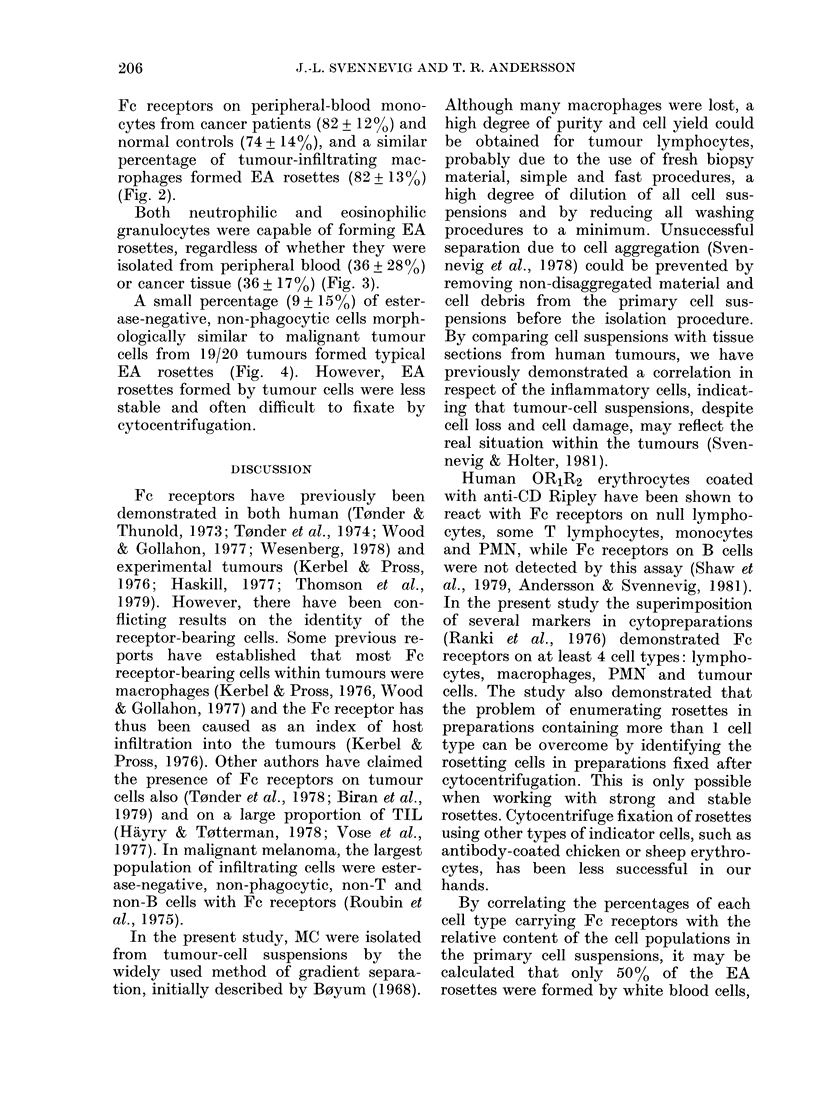

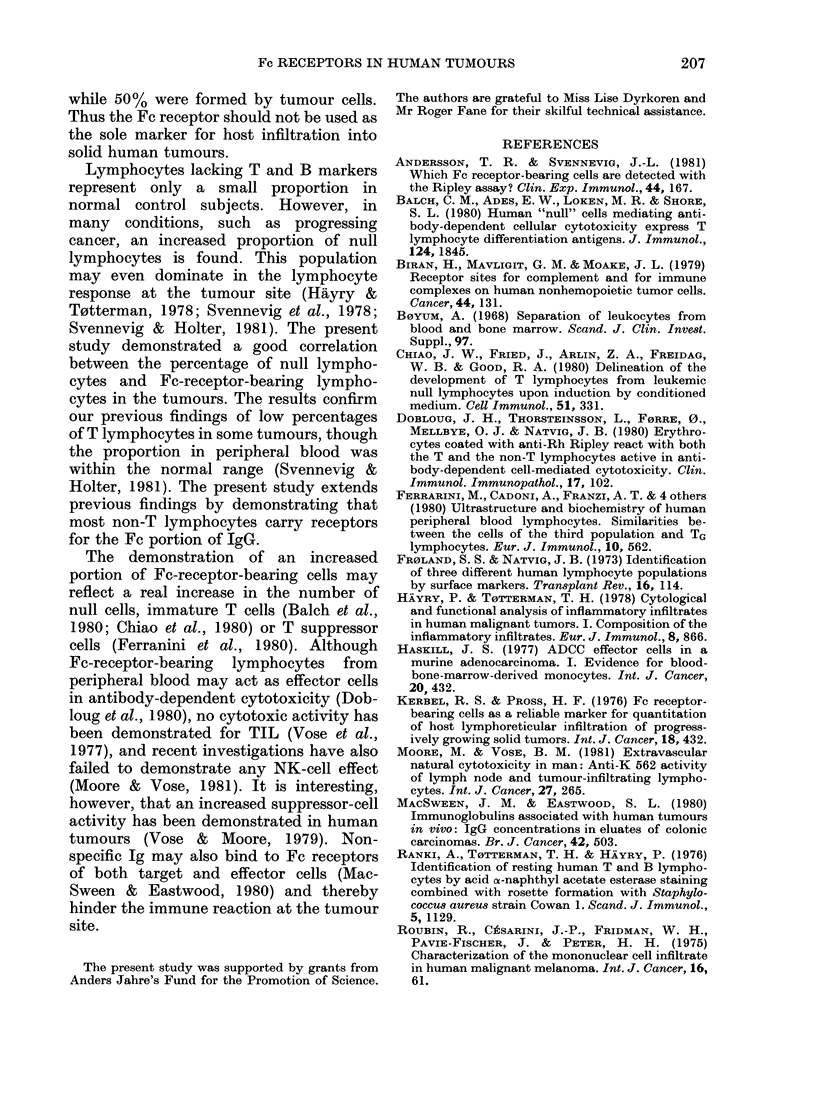

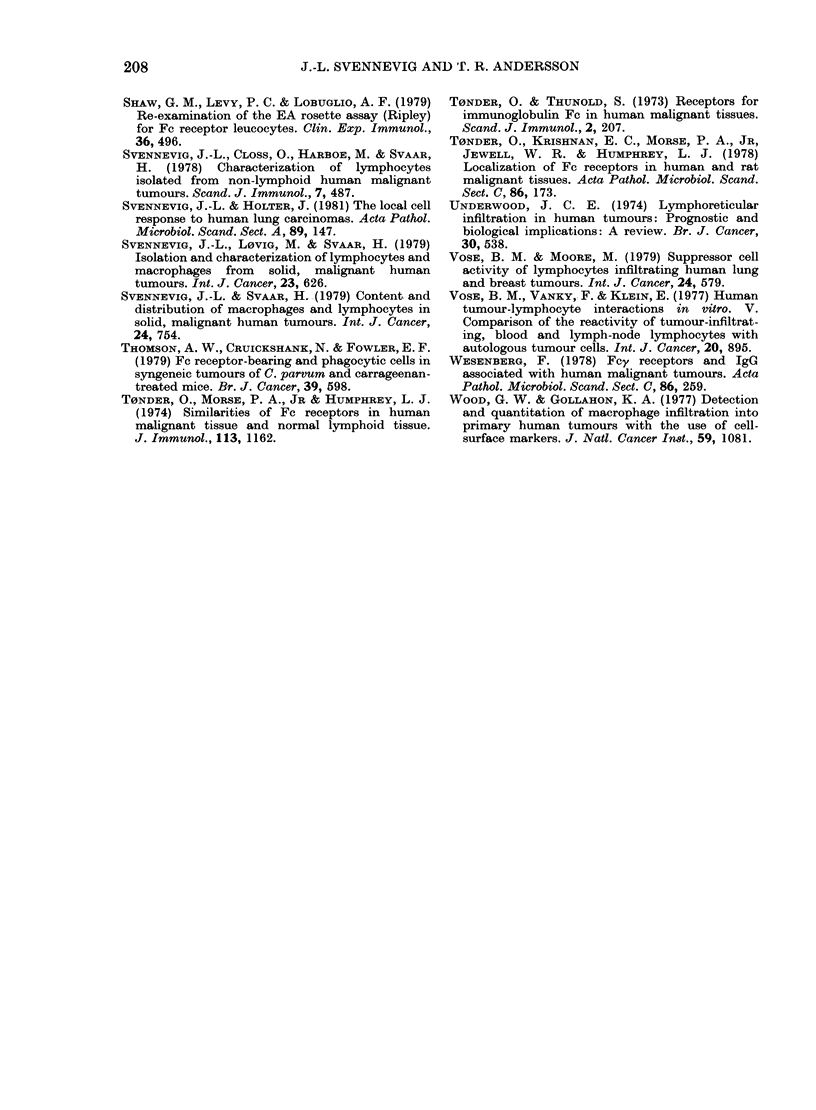


## References

[OCR_00570] Andersson T. R., Svennevig J. L. (1981). Which Fc receptor-bearing cells are detected with the Ripley assay?. Clin Exp Immunol.

[OCR_00575] Balch C. M., Ades E. W., Loken M. R., Shore S. L. (1980). Human "null" cells mediating antibody-dependent cellular cytotoxicity express T lymphocyte differentiation antigens.. J Immunol.

[OCR_00582] Biran H., Mavligit G. M., Moake J. L. (1979). Receptor sites for complement and for immune complexes on human nonhemopoietic tumor cells.. Cancer.

[OCR_00593] Chiao J. W., Fried J., Arlin Z. A., Freitag W. B., Good R. A. (1980). Delineation of the development of T lymphocytes from leukemic null lymphocytes upon induction by conditioned medium.. Cell Immunol.

[OCR_00600] Dobloug J. H., Thorsteinsson L., Førre O., Mellbye O. J., Natvig J. B. (1980). Erythrocytes coated with anti-Rh Ripley react with both the T and the non-T lymphocytes active in antibody-dependent cell-mediated cytotoxicity.. Clin Immunol Immunopathol.

[OCR_00608] Ferrarini M., Cadoni A., Franzi A. T., Ghigliotti C., Leprini A., Zicca A., Grossi C. E. (1980). Ultrastructure and cytochemistry of human peripheral blood lymphocytes. Similarities between the cells of the third population and TG lymphocytes.. Eur J Immunol.

[OCR_00615] Froland S. S., Natvig J. B. (1973). Identification of three different human lymphocyte populations by surface markers.. Transplant Rev.

[OCR_00625] Haskill J. S. (1977). ADCC effector cells in a murine adenocarcinoma. I. Evidence for blood-borne bone-marrow-derived monocytes.. Int J Cancer.

[OCR_00620] Häyry P., Tötterman T. H. (1978). Cytological and functional analysis of inflammatory infiltrates in human malignant tumors. I. Composition of the inflammatory infiltrates.. Eur J Immunol.

[OCR_00631] Kerbel R. S., Pross H. F. (1976). Fc receptor-bearing cells as a reliable marker for quantitation of host lymphoreticular infiltration of progressively growing solid tumors.. Int J Cancer.

[OCR_00642] MacSween J. M., Eastwood S. L. (1980). Immunoglobulins associated with human tumours in vivo: IgG concentrations in eluates of colonic carcinomas.. Br J Cancer.

[OCR_00636] Moore M., Vose B. M. (1981). Extravascular natural cytotoxicity in man: Anti-K562 activity of lymph-node and tumour-infiltrating lymphocytes.. Int J Cancer.

[OCR_00648] Ranki A., Tötterman T. H., Häyry P. (1976). Identification of resting human T and B lymphocytes by acid alpha-naphthyl acetate esterase staining combined with rosette formation with Staphylococcus aureus strain Cowan 1.. Scand J Immunol.

[OCR_00656] Roubin R., Césarini J. P., Fridman W. H., Pavie-Fischer J., Peter H. H. (1975). Characterization of the mononuclear cell infiltrate in human malignant melanoma.. Int J Cancer.

[OCR_00665] Shaw G. M., Levy P. C., Lobuglio A. F. (1979). Re-examination of the EA rosette assay (Ripley) for Fc receptor leucocytes.. Clin Exp Immunol.

[OCR_00671] Svennevig J. L., Closs O., Harboe M., Svaar H. (1978). Characterization of lymphocytes isolated from non-lymphoid human malignant tumours.. Scand J Immunol.

[OCR_00677] Svennevig J. L., Holter J. (1981). The local cell response to human lung carcinomas.. Acta Pathol Microbiol Scand A.

[OCR_00682] Svennevig J. L., Lövik M., Svaar H. (1979). Isolation and characterization of lymphocytes and macrophages from solid, malignant human tumours.. Int J Cancer.

[OCR_00688] Svennevig J. L., Svaar H. (1979). Content and distribution of macrophages and lymphocytes in solid malignant human tumours.. Int J Cancer.

[OCR_00694] Thomson A. W., Cruickshank N., Fowler E. F. (1979). Fc receptor-bearing and phagocytic cells in syngeneic tumours of C. parvum- and carrageenan-treated mice.. Br J Cancer.

[OCR_00700] Tonder O., Morse P. A., Humphrey L. J. (1974). Similarities of Fc receptors in human malignant tissue and normal lymphoid tissue.. J Immunol.

[OCR_00706] Tonder O., Thunold S. (1973). Receptors for immunoglobulin Fc in human malignant tissues.. Scand J Immunol.

[OCR_00711] Tönder O., Krishnan E. C., Morse P. A., Jewell W. R., Humphrey L. J. (1978). Localization of Fc receptors in human and rat malignant tissues.. Acta Pathol Microbiol Scand C.

[OCR_00718] Underwood J. C. (1974). Lymphoreticular infiltration in human tumours: prognostic and biological implications: a review.. Br J Cancer.

[OCR_00724] Vose B. M., Moore M. (1979). Suppressor cell activity of lymphocytes infiltrating human lung and breast tumours.. Int J Cancer.

[OCR_00729] Vose B. M., Vánky F., Klein E. (1977). Human tumour--lymphocyte interaction in vitro. V. Comparison of the reactivity of tumour-infiltrating, blood and lymph-node lymphocytes with autologous tumour cells.. Int J Cancer.

[OCR_00736] Wesenberg F. (1978). Fc gamma receptors and IgG associated with human malignant tumours.. Acta Pathol Microbiol Scand C.

[OCR_00741] Wood G. W., Gollahon K. A. (1977). Detection and quantitation of macrophage infiltration into primary human tumors with the use of cell-surface markers.. J Natl Cancer Inst.

